# Chronic Manganese Toxicity Associated with Voltage-Gated Potassium Channel Complex Antibodies in a Relapsing Neuropsychiatric Disorder

**DOI:** 10.3390/ijerph15040783

**Published:** 2018-04-18

**Authors:** Cyrus S.H. Ho, Roger C.M. Ho, Amy M.L. Quek

**Affiliations:** 1 Department of Psychological Medicine, Yong Loo Lin School of Medicine, National University of Singapore, Singapore 119074, Singapore; pcmrhcm@nus.edu.sg; 2 Division of Neurology, Department of Medicine, Yong Loo Lin School of Medicine, National University of Singapore, Singapore 119228, Singapore; Amy_QUEK@nuhs.edu.sg; 3 Division of Neurology, University Medicine Cluster, National University Hospital, Singapore 119074, Singapore

**Keywords:** manganese toxicity, voltage-gated potassium channel complex antibodies, neuropsychiatric disorder

## Abstract

Heavy metal poisoning is a rare but important cause of encephalopathy. Manganese (Mn) toxicity is especially rare in the modern world, and clinicians’ lack of recognition of its neuropsychiatric manifestations can lead to misdiagnosis and mismanagement. We describe the case of a man who presented with recurrent episodes of confusion, psychosis, dystonic limb movement and cognitive impairment and was initially diagnosed with anti-voltage-gated potassium channel (VGKC) complex limbic encephalitis in view of previous positive autoantibodies. His failure to respond to immunotherapy prompted testing for heavy metal poisoning, which was positive for Mn. This is the first report to examine an association between Mn and VGKC antibodies and the effects of Mn on functional brain activity using functional near-infrared spectroscopy (fNIRS).

## 1. Background

Manganese (Mn) is the twelfth most abundant element in the Earth’s crust [1] and is naturally found in soil, rocks, food and water. With its versatile chemical nature, Mn exists in various oxidation states, as well as salt and chelate forms. It is essential for normal cell growth and development and is involved in the mediation of the immune response, glucose regulation and metabolism of carbohydrates, lipids and proteins [2,3]. Mn serves as an activator or cofactor for many critical enzymes responsible for the eradication of reactive oxygen species (ROS), amino acid synthesis, urea production and energy synthesis [4,5]. Mn is also the fifth most abundant metal and fourth most commonly extracted metal [1], thanks to its role in industrial manufacturing of steel alloys, dry-cell batteries, paints, adhesives, gasoline, fertilizers, etc. Although Mn poisoning is not commonly reported in this modern era, heavy metal pollution in the context of increased industrial waste production increases the risk for metal poisoning. Occupational exposure via inhalation of Mn dust can occur in these settings and is believed to be the cause of the majority of cases of human toxicity cases. Inhaled Mn directly bypasses the blood-brain barrier and reaches the olfactory bulb of the brain, causing widespread neurotoxicity through a series of proposed mechanisms that have yet to be fully elucidated, which include the following: (1) Altered iron hemostasis: Mn directly competes with iron by binding with proteins and enzymes that require iron as a cofactor, and inhibition of iron-regulatory proteins leads to a compartmental shift in iron from the blood to the cerebrospinal fluid [6], resulting in excessive accumulation of iron in neurons, which leads to cellular oxidative stress and consequent neuronal damage; (2) Mitochondrial dysfunction: Inhibition of ATP synthesis in brain mitochondria leads to reduced intracellular ATP resulting in generation of free radicals and consequently mitochondrial dysfunction [7], thereby increasing overall oxidative stress, decreasing reductive capacity and leading to cytotoxicity and degeneration; (3) Dopamine oxidation and depletion: Oxidation of dopamine to reactive quinone species disrupts the mitochondrial membrane [8] and may contribute to dopaminergic cell death. Oxidative phosphorylation in dopaminergic neurons may impair presynaptic dopamine release, inhibit dopamine synaptic neurotransmission and deplete striatal dopamine, thereby resulting in motor deficits; (4) Cholinergic dysfunction: Binding of Mn to acetylcholinesterase (AChE) prevents the hydrolysis of AChE, leading to accumulation of acetylcholine (ACh) in the synaptic cleft and overstimulation of muscarinic and nicotinic ACh receptors [9], increasing oxidative stress, activating the cholinergic system, and further increasing neurotoxicity.

Neuropsychiatric symptoms of Mn toxicity are thought to arise from its accumulation in iron-rich regions of the basal ganglia and through an interaction between Mn and iron that leads to abnormal iron metabolism, it causes oxidative stress-induced neuronal damage [10]. The onset of poisoning is insidious, with symptoms gradually occurring months or years after exposure that can continue to progress more than 10 years later, even after Mn has been cleared from the body [11]. Initial neuropsychiatric features include fatigue and personality changes that could evolve into the development of hallucinations, delusions and hyperexcitability (previously described as locura manganica or ‘manganese madness’), and parkinsonism features (rigidity, flattened affect, action tremors, bradykinesia and gait disturbances) that can be accompanied by dystonia and dementia.

Mn accumulates in the basal ganglia of the brain, specifically in the globus pallidus, subthalamic nucleus, striatum and substantia nigra [12], which are involved in motor and non-motor functions, resulting in neuronal degeneration. Although the symptoms of Mn toxicity are similar to that of Parkinson’s disease, especially with the similar underlying pathophysiological mechanisms such as oxidative stress, excitotoxicity, mitochondrial dysfunction and cell death pathways, Mn toxicity can be distinguished by its lack of a therapeutic response to levodopa, less resting tremor and more frequent dystonia [13]. There is also a biphasic manner of deterioration with an initial psychiatric presentation before subsequent motor deficits in the latter phase of the disorder [12]. Long term, patients with Mn toxicity may show rapid deterioration in the first 5–10 years before reaching a plateau for the next 10 years, while those with Parkinson’s disease may deteriorate continuously [14]. The variation in symptomology stems from differences in the involvement of the brainstem between the two disorders. In Mn toxicity, GABAergic neurons in the globus pallidus are especially sensitive to degeneration, while the striatum is less affected [15], and other areas of involvement include the pons, cerebellum, thalamus, and anterior horn of the spinal cord [16]. Conversely, idiopathic Parkinson’s disease involves the specific degeneration of dopaminergic neurons within the nigra-striatal pathway of the substantial nigra pars compacta [17].

Heavy metals such as mercury, lead and cadmium are well known to be capable of causing autoimmunity by inhibiting immune cell proliferation and activation [18]. However, there has been no known association between Mn and voltage-gated potassium channel (VGKC) antibodies to the best of our knowledge. VGKCs are the most ubiquitous and diverse group of neuronal membrane-bound proteins present in both the central and peripheral nervous systems and mediate the repolarization of the nerve terminal after an action potential [19]. VGKC antibodies do not target the voltage-gated potassium channel directly but rather bind to associated proteins leucine-rich glioma-inactivated 1 (LGI1) or contactin-associated protein-like 2 (Caspr2), which have been found to predict differential clinical phenotypes [19]. While LGI1 antibody-positive patients commonly show severe cognitive impairment, confusion and faciobrachial dystonic seizures, Caspr2 antibody-positive patients may present with focal epilepsy, schizophrenia and, at high titers, can suffer from Morvan syndrome [20], which includes a spectrum of peripheral nerve hyperexcitability and neuropsychiatric features related to limbic encephalitis. However, VGKC-positive patients lacking LGI1 and Caspr2 antibodies show clinical heterogeneity, partly due to the lack of a distinct separation from those with associated proteins, which was only recognized eight years ago. A distinct feature that separates those with and without the associated proteins is that the presence of LGI1 or Caspr2 antibodies is associated with a better immunotherapy response [21], suggesting an inflammatory condition. Furthermore, VGKC-positive patients without the associated proteins did not show a better immunotherapy response than matched VGKC-negative patients [22], suggesting that VGKC positivity in the absence of LGI1 or Caspr2 antibodies might not be a marker of autoimmune inflammation.

Here, we illustrate a case of a man who was initially diagnosed with anti-VGKC complex limbic encephalitis and later found to have chronic Mn toxicity after four years. This is the first report to examine the association between Mn and VGKC antibodies and the effects of Mn on functional brain activity using functional near-infrared spectroscopy (fNIRS).

## 2. Case Report

In July 2016, a 55-year-old man presented with a four-day history of fluctuating consciousness, auditory and visual hallucinations, and delusions of persecution and poverty. Clinical examination was significant for flattened affect, stiffening and tensing of his limbs on tactile provocation without consistent rigidity. He had three similar presentations in the last four years, including the first where he developed dystonic limb movements and electrographic ictal activities on electroencephalogram (EEG), prompting a consideration for an immune etiology of his neurologic presentation. Anti-VGKC complex antibodies (181 pmol/L, normal < 100) were detected then, and a diagnosis of anti-VGKC complex limbic encephalitis was made. He was treated with intravenous methylprednisolone for his episodes and maintained on mycophenolate. Despite functional improvement and the ability to resume work, he suffered from mild cognitive impairment and was not able to actively engage in social activities. He had no prior personal or family history of psychiatric illness. His medical history was significant for chronic hepatitis B diagnosed in 2012, for which he was initially on azathioprine, which was subsequently stopped due to liver dysfunction secondary to treatment. He was switched to entecavir prophylaxis since 2014.

In this latest presentation, laboratory investigations were conducted to exclude potential infective, metabolic, endocrinological and rheumatological causes for his symptoms. The investigations were normal for full blood count, electrolytes, liver function, thyroid function, anti-double-stranded DNA antibodies, and antibodies to cardiolipin and extractable nuclear antigens. His cerebrospinal fluid (CSF) examination and brain magnetic resonance imaging (MRI) were also unremarkable, with the anti-VGKC complex antibody test being equivocal (86 pmol/L). Comprehensive re-assessment of neural-specific autoantibodies in serum and CSF, including Caspr2 and LGI1, were negative. EEG showed mild generalized encephalopathy. Whole-body positron emission tomography-computed tomography (PET-CT) scan was negative for malignancy. He was found to have unconjugated hyperbilirubinemia (35 µmol/L, normal < 25), and ultrasound of his hepatobiliary system revealed a distended gallbladder with sludge and possibly some wall thickening. The biliary tree was not dilated. The hepatologist was consulted, who assessed that there was no evidence of acute cholecystitis. The patient received intravenous methylprednisolone followed by intravenous immunoglobulin in addition to olanzapine and sodium valproate. Unlike his previous relapses, he showed no improvement with immunotherapy. His neurological condition evolved over the subsequent month with worsening stupor, diaphoresis, and fluctuating hypertonia with spurts of violent limb movement. A nasogastric tube had to be inserted due to dysphagia.

Additional tests to search for alternative causes of his presentation included a metabolic screen for homocysteine, amino acids, methylmalonic acid and organic acid, which were negative. A heavy metal screen that included mercury, lead, cadmium, arsenic and Mn was then performed. The blood test was negative for all heavy metals. However, the spot urine test was positive for Mn (62.11 µg/L, normal < 50), and the 24-h urine Mn measurement again revealed a significant elevation in Mn levels (19.7 mcg/24 h, normal < 4.0 mcg/24 h). Corroborative history from the family revealed a significant occupational history that accounted for his Mn exposure. Eight years prior, he had worked in a manufacturing plant in which he was exposed to polyurethane, polyethylene and polypropylene products for fifteen months, before working in a shipyard handling industrial sludge for a subsequent thirty-nine months. With the positive urinary Mn levels, significant occupational history and combination of neuropsychiatric symptoms comprising psychosis, cognitive impairment, parkinsonism and dystonia, he was diagnosed with chronic Mn toxicity.

**Figure 1:**
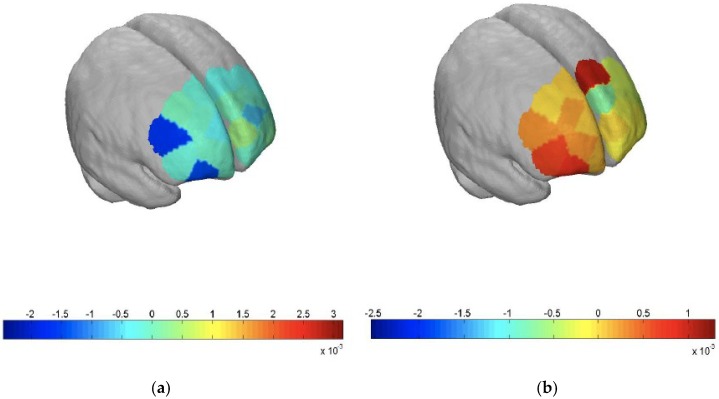
Functional near-infrared spectroscopy (fNIRS) imaging of the frontal lobe. The paradigm used in the imaging was the verbal fluency test (VFT), which required the individual to list as many words as possible that started with specific letters of the alphabet. The VFT is a short test of verbal functioning with involvement of the frontal lobe. The figure shows (**a**) areas of deactivation in the frontal lobe (highlighted in blue) in the patient compared with (**b**) areas of activation (highlighted in red) in an age- and gender- matched control, illustrating a frontal lobe impairment in chronic Mn toxicity.

By then, three months into his hospitalization, he had improved clinically. He was more alert and less agitated after an empirical trial of rivastigmine patch (9.5 mg/24 h) to ameliorate his rapidly progressive dementia. His mini-mental-state examination (MMSE) score was 17/30, and a formal neuropsychological assessment revealed severe impairments across all cognitive domains. He also underwent functional neuroimaging using fNIRS (NIRSport from LLC NIRx Medical Technologies, Minneapolis, MN, USA), which we believe is the first reported use of this technology in Mn toxicity (Figure 1a,b).

The scan revealed a pattern of deactivation in his frontal lobe while he performed the verbal fluency test paradigm compared to that in a healthy control, indicating executive dysfunction. On subsequent outpatient follow-ups, his cognition remained poor with parkinsonism features. Two months post-discharge, his MMSE score was 18/30. A repeat neuropsychological assessment done one-year post-discharge showed persistent global cognitive impairment. He had intermittent mild hand tremors with fine motor incoordination but was able to converse and walk independently. However, he was no longer able to work and had to be placed in a dementia day-care service.

## 3. Discussion

This is the first report of a patient with relapsing neuropsychiatric illness from chronic Mn toxicity associated with VGKC- complex antibodies. Our patient, in addition to a positive occupational exposure, had increased susceptibility to Mn toxicity in his latest admission likely due to deficient biliary excretion as evidenced by the raised unconjugated hyperbilirubinemia. He also had a background of chronic hepatitis B and was on immunosuppressants that could have affected his liver function. Studies have highlighted that deficits in biliary excretion resulting from liver injury or disease lead to elevated Mn levels in the blood and basal ganglia [23]. This is especially true in cases when biliary excretion is the predominant route of Mn excretion [2]. Currently, there are no reliable biomarkers to diagnose and evaluate the effect of Mn exposure on individuals due to a lack of understanding of the mechanisms of Mn toxicity. As a result, the diagnosis primarily depends on the combination of characteristic neuropsychiatric features and an occupational history of exposure [24]. As neither blood nor urinary Mn levels correlate with the severity of the neuropsychiatric manifestations, they are of limited value in the diagnosis other than confirming exposure or assisting in differential diagnosis. The relatively short biological half-life of Mn in the blood yet substantial accumulation in tissues, with a half-life of approximately eight–nine years expected in human bones [25], may allow the blood concentration to be elevated for only a very short period after Mn exposure, thereby making Mn blood levels unhelpful in the diagnosis of chronic toxicity and less relevant as an indicator of total body burden of Mn, which was the case for our patient. Only a very low percentage of Mn is excreted in the urine; thus, a high Mn level in the urine may assist in proving exposure. Although Mn toxicity classically produces bilateral T1 but not T2 signal hyperintensities in the basal ganglia (particularly the globus pallidus) on MRI imaging, this usually occurs in the acute stage and has been reported to resolve within six months to one year following the cessation of Mn exposure [26], as would be expected in chronic toxicity. ^1^H (proton) magnetic resonance spectroscopy (MRS) has been used to investigate levels of brain metabolites that could be used as neuronal integrity markers, and they are found to have correlations with Mn toxicity [27]. Significant increase in gamma-Aminobutyric acid (GABA) level in the thalamus combined with the pallidus index (defined as the ratio of signal intensity in the globus pallidus to that in the subcortical frontal white matter in axial T1-weighted MRI planes multiplied by 100) could potentially identify those with Mn exposure at the pre-symptomatic stage [28]. Furthermore, increase in glutamine and decrease in glutamate and N-acetylaspartate levels are found to correlate with Mn exposure [29]. Nevertheless, the wide spectrum of metabolite alterations as reported in various studies, likely due to differences in Mn-exposure settings, brain areas investigated, and different scan/analysis protocols, makes it challenging for comparison purposes.

The current inability of routine medical investigations to reveal abnormalities secondary to toxicity and the often non-specific signs and symptoms of neurotoxic damage, which can be easily confused with other conditions such as degenerative, infective, metabolic or psychiatric disorders, make the diagnosis and management all the more challenging. In this case, fNIRS was used as an adjunctive tool to illustrate the functional impairment in our patient. fNIRS is an innovative neuroimaging technique that has a similar mechanism of action to functional MRI (fMRI) [30], which exploits the different absorption spectra of oxygenated and deoxygenated hemoglobin in the near infrared region to measure alterations in the oxygenated (oxy-Hb) and deoxygenated (deoxy-Hb) hemoglobin concentrations, thereby reflecting regional cerebral blood flow. It is based on an underlying mechanism known as neurovascular coupling, whereby activation of a brain region is represented by an increase in oxy-Hb and a corresponding decrease in deoxy-Hb levels [31]. However, unlike fMRI, fNIRS allows us to conduct the scan in a naturalistic environment, is relatively insensitive to movement artifacts, and can be done quickly, which makes it more tolerable for our patient. It also has good temporal resolution of less than 1 s which is useful to delineate the time course of brain activity. Furthermore, it is less expensive than fMRI. Nevertheless, fNIRS only detects cerebral changes in the superficial cortex and does not elucidate the etiology of the dysfunction.

Treatment with *para*-aminosalicylic acid (PAS) chelation has been recommended for severe cases of poisoning to reduce the body burden of Mn and alleviate symptoms [32], but due to the rarity of cases, PAS may not be widely available, as illustrated in this case. Our patient was symptomatically treated with rivastigmine, and there has been some improvement in his agitation and attention. Nevertheless, no prior literature has elucidated the effects of AChE inhibitors in the treatment of Mn toxicity. We hypothesized that administration of a competitive antagonist to Mn (e.g., an AChE inhibitor) may momentarily engage the enzyme’s catalytic site by forming an unstable bond, thereby blocking the inhibition of the enzyme [33], which might account for our patient’s response to rivastigmine. Nevertheless, more research in the role and efficacy of AChE inhibitors in Mn toxicity is warranted.

Our patient was initially diagnosed with anti-VGKC complex limbic encephalitis due to the subacute onset of symptoms, VGKC complex seropositivity and an initial favorable steroid response. Subsequent VGKC complex antibody titers were reduced, and further serum and CSF examinations to evaluate for Caspr2 and LGI1 antibodies were negative. These latter antibodies, which target proteins associated with the VGKC complex, are considered to be more definitive markers of autoimmune inflammation [19]. However, the coincidental presence of VGKC complex antibodies in the setting of Mn toxicity and the patient’s initial improvement with immunotherapy raises the possibility that Mn toxicity-induced cell damage might trigger an autoimmune antibody response against the VGKC complex. Alternatively, the presence of the VGKC complex antibodies may have been a non-specific epiphenomenon, and his steroid response may have been due to a steroid-related acceleration in Mn distribution and excretion, as has been demonstrated in both animal and human studies [34].

This report highlights the complexity in the diagnosis of chronic Mn toxicity with its non-specific presentation, especially when there is a time lapse between exposure and symptom onset with no definitive biomarker of exposure. Furthermore, heavy metal testing is not a routine medical evaluation, and clinicians often have limited experience in the diagnosis and management of heavy metal poisoning. A high index of suspicion and a conscientious record of occupational history are essential. Our study also accentuates the importance of Mn toxicity prevention in occupational health, as no therapeutic option is viable with the progression of the clinical course once the neurodegeneration becomes permanent, even without further exposure. Lastly, there is a need to encompass a broad differential and consider a non-autoimmune trigger in patients who present with encephalopathy associated with anti-VGKC complex antibodies in the absence of Caspr2 or LGI1 antigenic specificity.
